# Reconstitution of Mammary Epithelial Morphogenesis by Murine Embryonic Stem Cells Undergoing Hematopoietic Stem Cell Differentiation

**DOI:** 10.1371/journal.pone.0009707

**Published:** 2010-03-15

**Authors:** Shuxian Jiang, Byeong-Chel Lee, Yigong Fu, Shalom Avraham, Bing Lim, Hava Karsenty Avraham

**Affiliations:** 1 Division of Experimental Medicine, Beth Israel Deaconess Medical Center and Harvard Medical School, Boston, Massachusetts, United States of America; 2 Hematology and Oncology, Beth Israel Deaconess Medical Center and Harvard Medical School, Boston, Massachusetts, United States of America; 3 University of Pittsburgh School of Medicine, Hillman Cancer Center, Pittsburgh, Pennsylvania, United States of America; 4 Stem Cell and Developmental Biology, Genome Institute of Singapore, Singapore, Singapore; City of Hope National Medical Center, United States of America

## Abstract

**Background:**

Mammary stem cells are maintained within specific microenvironments and recruited throughout lifetime to reconstitute *de novo* the mammary gland. Mammary stem cells have been isolated through the identification of specific cell surface markers and *in vivo* transplantation into cleared mammary fat pads. Accumulating evidence showed that during the reformation of mammary stem cell niches by dispersed epithelial cells in the context of the intact epithelium-free mammary stroma, non-mammary epithelial cells may be sequestered and reprogrammed to perform mammary epithelial cell functions and to adopt mammary epithelial characteristics during reconstruction of mammary epithelium in regenerating mammary tissue *in vivo*.

**Methodology/Principal Findings:**

To examine whether other types of progenitor cells are able to contribute to mammary branching morphogenesis, we examined the potential of murine embryonic stem (mES) cells, undergoing hematopoietic differentiation, to support mammary reconstitution *in vivo*. We observed that cells from day 14 embryoid bodies (EBs) under hematopoietic differentiation condition, but not supernatants derived from these cells, when transplanted into denuded mammary fat pads, were able to contribute to both the luminal and myoepithelial lineages in branching ductal structures resembling the ductal-alveolar architecture of the mammary tree. No teratomas were observed when these cells were transplanted *in vivo*.

**Conclusions/Significance:**

Our data provide evidence for the dominance of the tissue-specific mammary stem cell niche and its role in directing mES cells, undergoing hematopoietic differentiation, to reprogram into mammary epithelial cells and to promote mammary epithelial morphogenesis. These studies should also provide insights into regeneration of damaged mammary gland and the role of the mammary microenvironment in reprogramming cell fate.

## Introduction

Mammary gland development occurs mostly postnatally and is dependent on a complex interplay of systemic hormones and local growth factors [1], [2], [3]. The mammary gland is composed of a network of branching ducts that terminate in sac-like lobules embedded in stromal tissue. There are two primary epithelial cell lineages, the myoepithelial and the luminal cells (comprised of ductal and alveolar subtypes), which are presumed to arise from a common progenitor cell. Generation of a functional mammary gland from a single mammary stem cell has been reported by two groups [4], [5]. A discrete population of mouse mammary cells with cell-surface markers Lin^−^CD29hiCD24^+^ was found to be enriched for transplantable mammary stem cells [4]. It was also reported that a single cell, CD45^−^Ter119^−^CD31^−^Sca1^low^CD24^med^CD49f^high^, marked with a LacZ transgene, was able to reconstitute a complete mammary gland *in vivo*. The transplanted cells contributed to both the luminal and myoepithelial lineages and generated functional lobulo-alveolar units during pregnancy [4], [5].

The cleared fat-pad transplantation system provides a useful *in vivo* system for studying mammary epithelial morphogenesis [6]. This method is based on the fact that the mouse mammary gland is not fully developed at 3 weeks of age, making it possible to excise the rudimentary mouse mammary epithelium from the fat pad, resulting in a cleared fat pad devoid of any epithelium. Subsequent engraftment of mammary epithelial cells (MECs) before puberty yields an anatomically normal functional mammary gland.

The mammary microenvironment plays important function in the regeneration of mammary gland. Mammary stem cells are maintained within specific microenvironments (niches) in the mammary gland and these mammary stem cell niches can be reconstituted de novo by mammary epithelial cells as shown in several studies [7], [8], [9], [10], [11], [12], [13]. Transplanted epithelial cells from the glands can induce mammary regeneration by providing a niche with local signaling cells and extracellular matrix that sustain stem cells [14]. Additionally, parity-identified mammary epithelial cells (PI-MEC), originating from differentiating cells during pregnancy, were shown to possess pluripotency and self-renewal upon transplantation and contributed progeny directly to the formation of secretory acini upon subsequent pregnancies [11], [13]. Interestingly, a recent study [15] showed that testicular cells can interact with the mammary epithelial cells within the mammary stroma, proliferate and contribute to epithelial cell progeny, resulting in mammary outgrowth. These testicular cells, when mixed with mammary epithelial cells and when transplanted into denuded mammary fat pads, were able to adopt a mammary epithelial cell phenotype, indicating that the mammary niche can redirect spermatogenic cell fate into mammary epithelial cell fate *in vivo*. Further, murine fetal and adult neural stem cells (NSCs) when mixed with equal numbers of mammary epithelial cells (MECs) and inoculated into epithelium-divested mammary fat pads of prepubertal female mice, the NSCs contributed to mammary epithelial specific progeny and mammary outgrowths (16). Thus, non-mammary tissues could be altered from their initial cell fate lineage to adopt mammary epithelial characteristics upon interaction with mammary epithelial cells during reconstruction of mammary epithelium in regenerating mammary tissue in vivo (17). However, it is unknown whether other sources of stem cells can generate epithelial progenitors that can reconstitute mammary tissues without the contribution of MECs. Here, in this study, we examined whether murine embryonic stem (mES) cells, undergoing hematopoietic differentiation, can support the reconstitution of mammary outgrowth when transplanted into epithelium-divested mammary fat pads.

Embryonic stem (ES) cells are pluripotent cells derived from the inner cell mass [16], [17]. These cells are capable of undergoing an unlimited number of symmetrical divisions without differentiation. In addition, these cells can give rise to differentiated cell types of all three primary germ layers of the embryo [18]. To address whether mES cells can contribute to mammary epithelial morphogenesis, we established three-dimensional (3-D) cultures of mES, using E14, E14-GFP and Rosa26.6 ES cell lines, for differentiation into MECs. E14-GFP is a mouse ES cell line with green fluorescence protein (GFP) and the Rosa26.6 cells contain a LacZ, which was used for cell lineage tracing. In addition, we analyzed the potential of mES cells undergoing hematopoietic stem cell differentiation for their ability to induce mammary epithelial morphogenesis in vivo in mice and established the conditions for such reprogramming of cell fate in the mammary microenvironment.

## Results

### The Potential of Murine ES Cells to Differentiate Into Mammary Epithelial Cells

**Figure 1:**
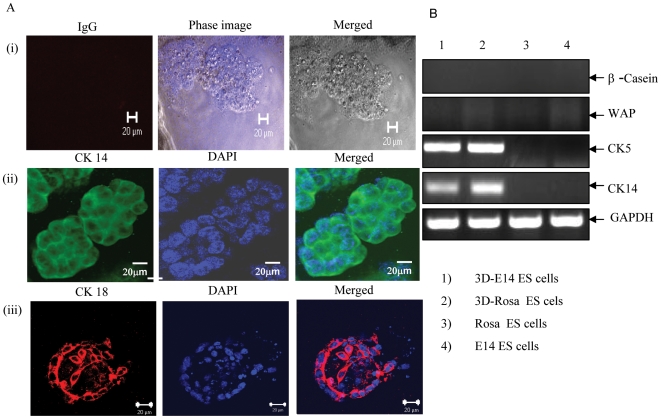
mES cells were able to clonally reproduce acini *in vitro.* (A) Confocal microscopy of mES E14-GFP cells (Panels A(i) and A(ii) and Rosa 26.6 ES cells Panel A(iii) showing acini structures. The mES cells were grown in epithelial media under 3D Matrigel matrix conditions. Cells were grown for 10 days and stained with control IgG (Panel i) or with CK14 antibody (Panel ii), or with cytokeratin CK 18 (Panel iii). In addition, nuclear staining (DAPI) was performed, and is shown at 60X magnification (scale bar, 20 µm). This is a representative experiment out of four experiments. (B) RT-PCR analysis of 3-D mammary epithelial cell cultures derived from 3D-E14-ES cells (lane 1) 3D-Rosa 26.6 mES cells (lane2), control Rosa ES cells and E14-ES cells, respectively (lanes 3 and 4). Specific primers for murine β-casein, WAP, CK 5, CK 14 and GAPDH were used in this analysis. This is a representative experiment out of 3.

To assess the multilineage differentiation potential of mES cells to generate *in vitro* complex functional structures reminiscent of the mammary tree, we employed the 3-D cultivation system which provides the physiological signal necessary for mammary morphogenesis *in vitro* and enables mammary cells to recapitulate the spatial orientation and architecture of the mammary tree *in vivo* [11], [13]. The differentiation potential of mES cells was assessed by determining the ability of mES cells to develop functional ductal-alveolar-like structures using Matrigel matrix. Acini structures were generated *in vitro* by mES cells, the E14-GFP and Rosa 26.6 ES cells, when cultured in epithelial media under 3D-culture conditions (Figure 1A). Nuclear staining and confocal microscopy showed elongated hollow tubular structures (Figure 1A, panels ii-iii). Next, these cells were tested for expression of the mammary gland cell-lineage specific markers CK5, CK14, WAP and β-casein. These cells were positive for CK5 and CK14, but negative for β-casein and for the specific mammary whey acidic protein (WAP) (Figure 1B). When these 3D mammary epithelial cells were examined for their potential to support mammary branching morphogenesis *in vivo*, these cells failed to differentiate *in vivo* along all three mammary gland cell lineages and were unable to structurally and functionally recapitulate the architecture of the mammary gland.

### Hematopoietic differentiation of murine ES cells and their transplantation into denuded mammary fat pads

Next, we examined the potential of embryoid bodies (EBs) derived from GFP-E14 mES cells to reconstitute mammary epithelial morphogenesis. First, we used EBs formed in medium without specific growth factors to allow random differentiation of ES cells. Transplantation of these EBs failed to induce mammary epithelial branching morphogenesis. Next, we employed EBs formed under hematopoietic differentiation conditions following established protocols (StemCell Technologies, Vancouver, Canada) involving a two-step EB method. First, spherical cell EB aggregates were generated that contain ectodermal, mesodermal and endodermal derivatives (Primary Differentiation). EB aggregates were then selected for hematopoietic precursors and expanded with growth factors consisting of EPO, IL-3 and IL-6 (Secondary Hematopoietic Differentiation). Cells were processed for the Wright-Giemsa staining; RT-PCR and Western blot experiments at different times of EB culture differentiation as indicated.

**Figure 2:**
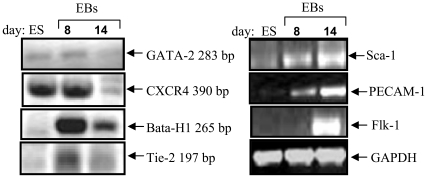
Time course of hematopoietic differentiation of mES-E14/GFP. Differentiated hematopoietic EBs at days 8 and 14 were analyzed by RT-PCR analysis. This is a representative experiment out of 5.

**Figure 3:**
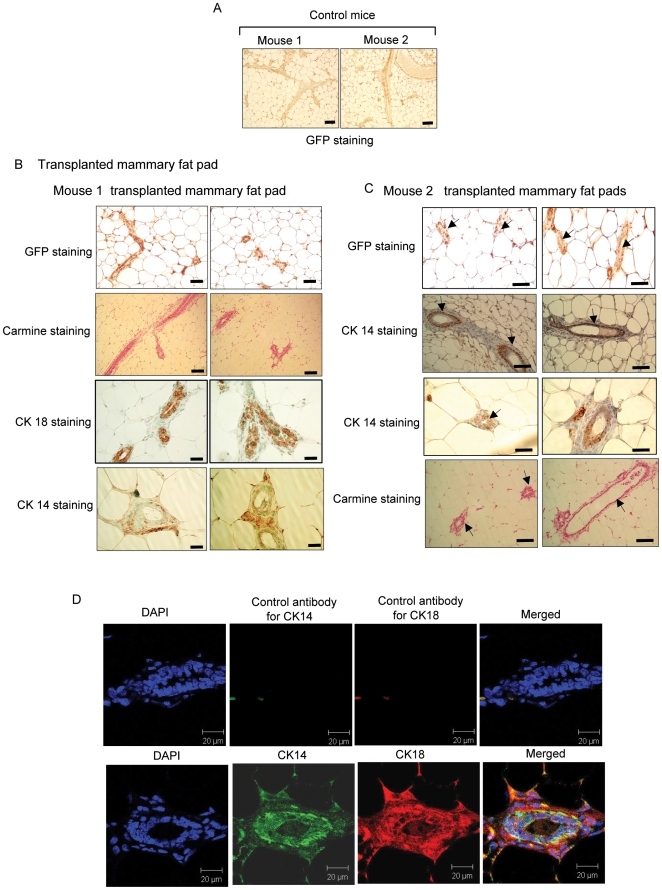
Hematopoietic EB-derived E14 (GFP) mES cells at day 14 can reconstitute mammary fat pads. (A) Sections of WT mammary fat pads derived from 2 control mice stained with anti-GFP antibody. This served as negative control for GFP staining (20X magnification; scale bar, 100 µm). (B-C) Sections of GFP+ outgrowths derived from reconstituted mammary fat pads from mice stained with GFP, carmine, CK 14 and CK 18 as indicated. 20× magnification for GFP and Carmine staining. B: (scale bar, 100 µm), 40X magnification for CK18 and CK14 staining (scale bar, 50 µm) C: 20X magnification (scale bar, 100 µm). These are representative analyses of immunostaining of transplanted mammary fat pads, out of 56 transplanted mammary fat pads. (D) Reconstituted mouse mammary epithelial cells stained for either control antibodies or for both CK 18 (red) and CK 14 (green). 4′,6-diamidino-2-phenylindole (DAPI) (blue) was used to stain the nuclei. 1:300 diluted polyclonal antibody of CK 14 (Covance, Catalogue number PRB-155p) and 1:300 diluted monoclonal antibody of CK 18 were used for immunostaining. Secondary antibodies were added and sections were imaged using a confocal microscope (60X magnification; scale bar, 20 µm).

**Figure 4:**
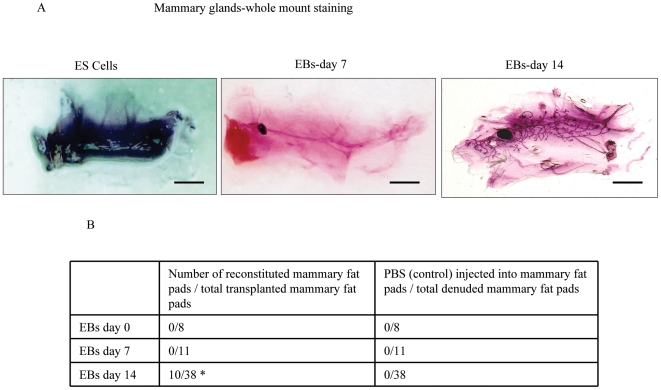
Reconstitution of mammary epithelium by mES cells. (A) Whole-mount staining of transplanted mammary glands. The 4^th^ mammary fat pad of 3 weeks old female mice were denuded and transplanted with either E14 mES cells, or EBs at day 7 or EBs at day 14, (ES cells were *in-vitro* differentiated in Methylcellulose into hematopoietic EBs at day 14 or at day 7), as indicated. Eight weeks after transplantation, whole-mount staining of the mammary fat pads were performed with carmine staining (magnification 2X, scale bar: 10 mm). (B) Virgin recipients harvested 8 weeks after transplantation with hematopoietic EB-derived E14-GFP ES cells at days 0, 7 and 14, as indicated. As a control, the mammary fat pad was denuded and injected with PBS at the same time and under the same conditions as the other samples. After 8 weeks of transplantation, the mammary fat pads were analyzed with carmine staining, GFP staining, CK 14, CK 18 and by whole mount analysis of epithelial outgrowths arising from transplantation. Controls were mammary fat pads from denuded mice without transplantation. *Statistical significance p<0.005.

Expression of hematopoietic specific markers, such as Bata-H1, Tie-2, Flk-1, Sca-1 and PECAM-1 were analyzed in EBs. As expected (Figure 2), hematopoietic-differentiated EBs derived from GFP-E14 mES cells at day 8 and day 14 expressed Bata- H1/Sca1^+^/Flk1^+^/PECAM1^+^. These EB-derived from GFP-E14 mES cells were then transplanted into denuded mammary fat pads. Substantial engraftment of the fat pad by EBs at day 14 was evident (Figures 3–4).

Histological sectioning of the mammary glands revealed normal ductal structures composed of both luminal and myoepithelial cells, as indicated by carmine staining and immunostaining with specific luminal marker cytokeratin 18 (CK18) or the myoepithelial marker cytokeratin 14 (CK14) (Figure 3B, 3C, 3D). We found that while EBs at day 7 were unable to support mammary outgrowths, the EBs at day 14 undergoing hematopoietic differentiation, contributed to the reconstitution of ∼26% of the mammary fat pads. The contribution of mammary morphogenesis by day 14 EBs derived from GFP-E14 mES cells, resulted in functional reconstitution of the mammary tissue as revealed by Carmine analysis, GFP expression, and expression of cytokeratin 14 and 18 at 8 weeks post transplantation (Figure 3B, 3C).

Reconstitution was observed in 10 denuded mice out of 38 mice transplanted with GFP-E14 mES cells, while no reconstitution was observed in denuded mice transplanted with either GFP-E14-EBs at day 7 or at day 0 (Figure 4A, 4B). Importantly, we did not observe any reconstitution of denuded mammary fat pads injected with PBS (total of 57), indicating that tfat pads were properly cleared (Figure 4).

**Figure 5:**
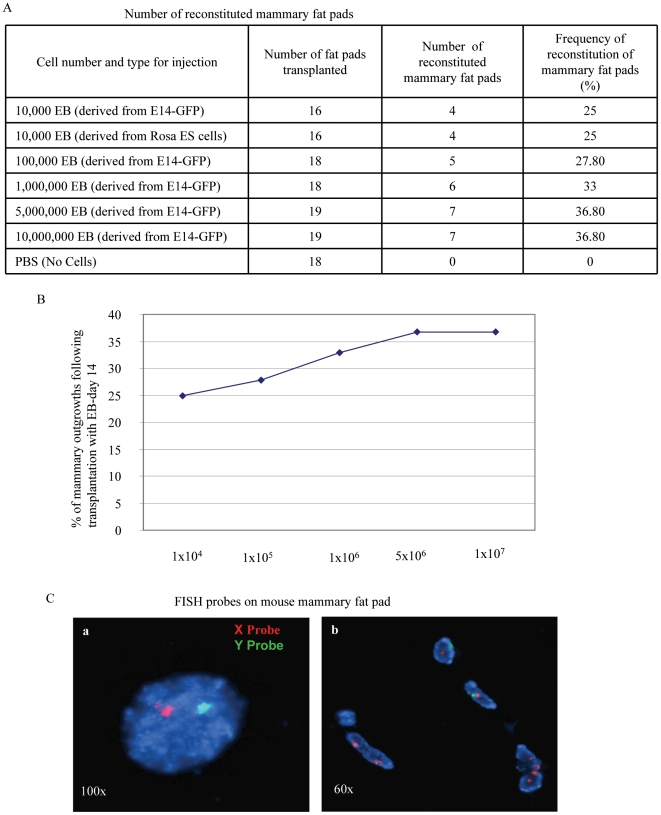
Effects of increasing numbers of hematopoietic EB-derived mES cells on mammary epithelial morphogenesis. (A) Immunostaining and whole-mount analysis of epithelial outgrowths induced bytransplantation of day14 EBs. (B) The mammary fat pads were analyzed with carmine staining, GFP staining, CK 14, CK 18 and by whole mount analysis of epithelial outgrowths arising from transplantation. (C) FISH with mouse X and Y chromosome probes on reconstituted mammary gland. Mouse X and Y chromosome FISH analysis were used on paraffin-embedded sections of reconstituted mammary glands with mEBs derived from E14-GFP mES cells at day 14. The X chromosome was labeled in red and the Y chromosome (detecting mES cells derived EBs) was labeled in green (y). The nuclei were stained with DAPI. a. Positive mammary epithelial cell for both x and y probes (×100 magnification); b. Positive mammary epithelial cells for both x and y probes are shown, as detected in the duct sections of the reconstituted mammary fat pads with EBs at day 14 (×60 magnification).

Examination of the mammary fat pads for GFP expression (for EBs derived from E14-GFP) or with LacZ staining (for EBs derived from Rosa26.6 ES cells) 8 weeks after injection clearly demonstrated sites of engraftment and expansion of these EB cells in ∼25% of the total inoculated fat pads (Figure 5A). In addition, in order to estimate the efficiency of reconstitution, we performed a dose response by increasing the number of transplanted day-14 EB-derived cells from 10^5^ to 10^6^, 5×10^6^ and 10^7^. Transplantation of these EB-derived cells resulted in an enhanced number of reconstituted mammary morphogenesis (Figure 5A, 5B). Of note, we did not observe any cases of teratoma formation when cells from day 7 or 14 EBs were transplanted into mammary fat pads (a total of more than 120 transplantation of mammary fat pads were performed), while mES cells without differentiation formed teratoma in vivo (data not shown).

### Supernatants derived from EBs failed to support mammary reconstitution

To further assess the importance of the contribution of cells from murine hematopoietic EBs to the reconstitution of mammary branching morphogenesis, denuded mammary fat pads were injected with supernatants derived from EBs at days 7 and 14. While the reconstitution of mammary epithelial morphogenesis was observed with day-14 EB cells (as revealed by carmine staining, GFP expression and expression of cytokeratins 14 and 18 at 8 weeks post transplantation), supernatants generated with day-7 and day-14 EBs injected into denuded mammary fat pads (n = 20) failed to induce the reconstitution of mammary epithelial morphogenesis (data not shown). These results indicate that the presence of cells from hematopoietic differentiated murine EBs contributing progenitor cells for mammary outgrowths are needed in the denuded mammary fat pads in order to interact with the stroma microenvironment within the mammary fat pads and to contribute to the reconstitution de novo mammary outgrowths.

### Demonstration of Y chromosome-specific sequences in mammary glands

PCR analysis of the DNA isolated from the reconstituted mammary outgrowths demonstrated the presence of sequences specific to the Y chromosome, verifying the presence of the donor murine ES cell DNA (Figure 5C). Positive cells were detected for the Y chromosome, suggesting that hematopoietic EBs derived from mouse ES cells contribute epithelial cell progeny and resulting in mammary outgrowths.

## Discussion

While all previous reports had demonstrated the reconstitution of mammary morphogenesis using mammary epithelial progenitor cells, this study provides the first description, to our knowledge, of the reconstitution of the mammary morphogenesis using cells that were not derived from primary mammary tissues. Here we showed that mammary morphogenesis can be induced and promoted by transplantation of murine ES cells undergoing hematopoietic differentiation. The transplanted EB-derived cells were able to contribute to branching duct-like structures that recapitulate the ductal-alveolar-like architecture of the mammary tree, providing support to both the luminal and myoepithelial lineages. We did not observe teratoma formation when these cells were transplanted *in vivo*. These results strongly suggest the importance of the mammary gland microenvironment for the generation of breast epithelial cells, derived from murine ES cells undergoing hematopoietic differentiation.

The interaction of epithelial cells with the stromal cells in the mammary gland is critical for mammary gland development and the differentiation into luminal and myoepithelial cell lineages. The epithelial tissue-specific stem cells are dispersed throughout the mammary gland and are maintained in the surrounding microenvironment. A parity-induced mammary epithelial cell population (PI-MEC) that possesses the properties of pluripotency and self-renewal upon transplantation was shown to reconstitute mammary outgrowth and morphogenesis [15]. Recently, epithelial cells derived from testicular cells, when supplied with mammary epithelial cells [15], were shown to alter their cell fate upon interaction with the mammary gland microenvironment during pregnancy, lactation and involution. These testicular cells were able to interact with the mammary epithelial cells within the stroma, proliferate and contribute epithelial cell progeny to the resulting mammary outgrowth. Here, we extended these observations and showed that murine hematopoietic cells derived from mES cells were also able to reconstitute of mammary morphogenesis.

These studies support the notion that the mammary tissue microenvironment has a critical role in reprogramming the intrinsic nature of cells from an alternative adult tissue and can redirect stem cell fate when transplanted into mammary fat pad. Our experimental data showed that mES undergoing hematopoietic differentiation can directly reprogram to epithelial progeny and de novo regeneration of stem cell niches in the mammary glands based on the following data:

1. In all control experiments where denuded fat pads had sham injection of PBS and no exogenous cells, we never observed any reconstitution of mammary gland.
2. No reconstitution was observed when murine cells from embryoid bodies undergoing random differentiation were transplanted. Reconstitution was observed only when murine cells were used from EB bodies generated under conditions that promote hematopoietic differentiation.
3. Reconstitution from transplanted murine cells was further supported by the use of GFP-tagged ES cell line. GFP+ cells were observed in branching mammary cells that stained also for carmine and other specific marker for luminal (CK18) and myoepithelial (CK14) cytokeratin.

Taken together, the study provides clear evidence for the ability of differentiated murine ES cells enriched for hematopoietic progenitor cells to regenerate mammary morphogenesis *in vivo*. The study provides clear evidence for the critical role of the mammary microenvironment in determining progenitor cell fate and mammary cell function. The assay we described is useful for studying “mammary morphogenesis” with potential application as a possible model system for drug discovery.

## Materials and Methods

### E14 differentiation to epithelial cells in 3D culture

Growth Factor Reduced Matrigel was purchased from BD Biosciences (354230). Fifty µl of growth factor reduced matrix gel was added to each well chamber. The chambers were placed in a cell culture incubator to allow the basement membrane to solidify for at least 20 minutes. While the Matrigel was solidifying, ES cells were trypsinized and single cells were seeded on Growth Factor Reduced Matrigel. The cells were grown in mammary epithelial cell media purchased from Clonetics (CC-4136), containing 2% FBS and 2% Matrigel. Every two to three days, the media were replaced with fresh media. The cells formed clusters by day 4–6 of the 3-D cultures and subsequently started forming hollow lumen [19]. Cells were then analyzed for specific mammary epithelial cell markers by confocal microscopy and RT-PCR methods.

### Derivation of embryoid bodies from ES cells

The Rosa26.6 ES cell line was obtained from Dr. Stuart Orkin (Children's Hospital, Harvard Medical School); The E14 and GFP-E14 cell lines were obtained from Dr. Bing Lim (Beth Israel Deaconess Medical Center, Boston). Culture and maintenance of ES cells in an undifferentiated state were performed as described previously [20]. Briefly, ES cells were maintained on a mouse Primary Embryonic Fibroblast (PEF) feeder cell line in ES medium containing Dulbecco's modified Eagle's medium (DMEM) with high glucose, 10 ng/ml murine leukemia inhibitory factor (mLIF; Chemicon International, Temecula, CA), 15% fetal calf serum (FCS; Hyclone, Logan, UT), 1 mM sodium pyruvate, 2 mM glutamine, 0.1 mM nonessential amino acids, 100 µM monothioglycerol (MTG; Sigma), 50 U/ml penicillin, and 10 µg/ml streptomycin. The ES cell lines were regularly analyzed by using an ES cell characterization kit (Chemicon), for determination of alkaline phosphatase activity and detection of surface markers and transcription factors that are expressed by undifferentiated ES cells, such as Oct-4, Rex-1, SSEA-1 and FoxD3 (Genesis).

*In vitro* hematopoietic differentiation of ES cells was performed as described, essentially according to the protocol of StemCell Technologies. The embryoid body (EB) method involves two steps: first, spherical cell aggregates (termed embryoid bodies = EBs) are generated that contain ectodermal, mesodermal and endodermal derivatives ( = Primary Differentiation); second, these aggregates are selected for hematopoietic precursors and expanded with growth factors such as EPO, IL-3 and IL-6 ( = Secondary Hematopoietic Differentiation). Briefly, EBs were generated in 1% methylcellulose cultures (1×10^4^ ES cells per 35 mm Petri dish). To promote primary differentiation into EBs, ES cells were cultured in ES differentiation medium containing Iscove's modified Dulbecco's medium (IMDM), 15% FBS, 2 mM L-glutamine, 150 µM MTG, and 40 ng/ml mSCF. After 8 days of differentiation, the EBs were collected and washed. 1×10^4^ of single cells were seeded on 1% methylcellulose from the secondary hematopoietic differentiation medium. 15% FBS, 2 mM L-glutamate, 150 µM MTG, 20% BIT (10% BSA, 10 µg/ml insulin, 200 µg/ml transferrin), 150 ng/ml mSCF, 30 µg/ml IL-3, 30 µg/ml IL-6 and 3 U/ml Epo were added to the culture to promote hematopoietic differentiation. Cells were processed for Wright-Giemsa staining, RT-PCR and Western blot analyses at different times of EB culture differentiation, as indicated.

To determine the characteristics of various types of hematopoietic progenitors present during ES cell differentiation, EBs from ES cell lines were collected from the cultures at days 8 and 14 (from the day of replating) to obtain the hematopoietic progenitors. Following cytospin preparation of these cells, they were stained with Wright-Giemsa and examined under a light microscope. Undifferentiated ES cells have a large nucleus, minimal cytoplasm, and one or more prominent dark nucleoli. Hematopoietic progenitors found in EB-day 14 cultures were identified by the morphology of erythroids, megakaryocytes, monocytes/macrophages, granulocytes and mast cells, as analyzed by brightfield microscopy.

### Mammary Fat Pad Transplantation and Analysis

Murine cells were resuspended in PBS and into the inguinal mammary glands of 3-week-old female BALB/c mice that had been cleared of endogenous epithelium. All mice were housed in AAALAC-accredited facilities in accordance with the NIH Guide for the Care and Use of Laboratory Animals. The BIDMC and Harvard Medical School Committees approved all experimental procedures. Recipient glands were removed for evaluation after 8 weeks unless otherwise stated. Whole mounts of wild-type mammary outgrowths were stained with hematoxylin. LacZ^+^ outgrowths were detected by X-Gal staining. GFP^+^ outgrowths were detected by GFP staining. An outgrowth was defined as an epithelial structure comprised of ducts arising from a central point, with lobules and/or terminal end buds.

### Immunostaining

Paraffin-embedded sections were deparaffinized and dehydrated, washed in PBS, and subjected to antigen retrieval using treatment with 150 mM glycine, microwaved for 15 minutes, before blocking. Primary antibody staining was performed overnight at 4°C, while secondary antibody staining was performed for 30 minutes and DAPI staining for 5 minutes at room temperature. Antibodies for GFP, cytokeratin 14 (Covance) and cytokeratin 18 (Progen Biotechnik) were used for immunohistochemistry. Fluorochrome-conjugated secondary antibodies included anti-rabbit and anti-mouse Ig-Alexa_594_ and anti-rabbit Ig-Alexa_488_ (Molecular Probes). Matrigel cultures were prepared and immunostained as described [21]. Sections were imaged using a confocal microscope (Zeiss LAM 510Meta). For human and murine adult hematopoietic stem cell transplantation, the paraffin-embedded sections were immunostained with human (cytokeratin Pan) (ABR Catalog #MA1-26268) and murine (cytokeratin 18 and 14) (Zymed Laboratories).

### Whole Mounts and Immunohistochemistry

For murine whole-mount analysis, fresh mammary tissue was flattened and fixed in Carnoy's solution (ethanol, glacial acetic acid, and chloroform) and subsequently stained with carmine alum (carmine, AlKSO_4_).

### Differentiation Culture Conditions, Cell Fixation, Staining, and Confocal Microscopy Analysis

Cells were washed with 1×PBS, fixed for 10 minutes with 4% paraformaldehyde-PBS, and then stained for CK18 and/or CK14, as indicated. Confocal microscopy analysis was performed using a Zeiss LAM 510Meta.

### LacZ Staining in Whole Mammary Tissue

The bacterial beta-galactosidate gene lacZ is frequently used as a reporter gene. The expression of transgenic constructs can be monitored by histochemistry with the chromogenic substrate X-gal. This allows precise cellular localization of gene activity. No background staining was detectable in mammary tissue when lacZ staining was performed. The mammary gland tissue was fixed for 2 hours in 2% paraformaldehyde, 0.25% glutaraldehyde, 0.01% NP-40 in PBS. Slides were rinsed and incubated with PBS containing 2 mM MgCl_2_, 0.01% Na-deoxycholate, and 0.02% NP-40. The samples were incubated for 2 hours, followed by addition of X-gal staining buffer containing 1 mg/ml X-gal. The staining buffer contained: 30 mM K_4_Fe(CN)_6_, 30 mM K_3_Fe(CN)_6_.3H_2_O, 2 mM MgCl_2_, 0.01% Na-desoxycholate, 0.02% NP-40, 1×PBS. The samples were then incubated at 30°C for 24 hours and analyzed under the microscope.

### FISH Analysis

One µg of mouse Cot-1 DNA (Invitrogen, USA) was labeled with SpectrumGreen-dUTP (Vysis Inc, USA), using nick translation kit (Abbott Molecular Inc., USA). Labeled Cot 1 DNA was ethanol precipitated and reconstituted by hybridization buffer (50% formamide, 2XSSC, 50 mM phosphate buffer, 10% dextran sulfate). Mouse X and Y probes used in this study were chromosome paints (X Cy3 labeled and Y FITC labeled) from Cambio, UK. For paraffin tissue section, the slides were initially incubated at 56°C overnight, then deparaffinized in Xylene, dehydrated in 100% ethanol and air dried, followed by incubation in TE at 100°C for 45 minutes. The slides were then treated with 150 µl of ZYMED Digest-All (Invitrogen, USA) under a plastic coverslip at 37°C for 20 minutes, followed by rinsing in PBS and dehydration in ethanol series. Two hundred ng of each probe (total volume 8 µl) were placed onto the area under a 22×22 mm coverslip and sealed with rubber cement. The slides with probe were denatured at 95°C for 3 minutes and hybridization was performed in a moist chamber at 37°C for 48 hours. Hybridized slides were washed in 0.5×SSC at 72°C for 5 minutes, in 0.025% Tween/PBS at RT for 2 minutes and dehydrated in ethanol series. Finally, the slides were mounted with 12 µl of DAPI II (Abbott Molecular Inc., USA). FISH slides were analyzed using an Olympus microscope. Images were taken by using a CCD camera and CytoVision software (Applied Imaging, Inc).

### Statistical analysis

Statistical differences for the number of mammary outgrowths in denuded mammary fat pads were determined using Student's t-test.

## References

[1] Hennighausen L, Robinson GW (1998). Think globally, act locally: the making of a mouse mammary gland.. Genes Dev.

[2] Daniel CW, Smith GH (1999). The mammary gland: a model for development.. J Mammary Gland Biol Neoplasia.

[3] Smith GH (2005). Label-retaining epithelial cells in mouse mammary gland divide asymmetrically and retain their template DNA strands.. Development.

[4] Shackleton M, Vaillant F, Simpson KJ, Stingl J, Smyth GK (2006). Generation of a functional mammary gland from a single stem cell.. Nature.

[5] Stingl J, Eirew P, Ricketson I, Shackleton M, Vaillant F (2006). Purification and unique properties of mammary epithelial stem cells.. Nature.

[6] Deome KB, Faulkin LJ, Bern HA, Blair PB (1959). Development of mammary tumors from hyperplastic alveolar nodules transplanted into gland-free mammary fat pads of female C3H mice.. Cancer Res.

[7] Daniel CW, De Ome KB, Young JT, Blair PB, Faulkin LJ (1968). The in vivo life span of normal and preneoplastic mouse mammary glands: a serial transplantation study.. Proc Natl Acad Sci U S A.

[8] Daniel CW, Young LJ (1971). Influence of cell division on an aging process. Life span of mouse mammary epithelium during serial propagation in vivo.. Exp Cell Res.

[9] Young LJ, Medina D, DeOme KB, Daniel CW (1971). The influence of host and tissue age on life span and growth rate of serially transplanted mouse mammary gland.. Exp Gerontol.

[10] Daniel CW, Deome KB (1965). Growth of Mouse Mammary Glands in Vivo after Monolayer Culture.. Science.

[11] Smith GH (1996). Experimental mammary epithelial morphogenesis in an in vivo model: evidence for distinct cellular progenitors of the ductal and lobular phenotype.. Breast Cancer Res Treat.

[12] Smith GH, Gallahan D, Zwiebel JA, Freeman SM, Bassin RH (1991). Long-term in vivo expression of genes introduced by retrovirus-mediated transfer into mammary epithelial cells.. J Virol.

[13] Kordon EC, Smith GH (1998). An entire functional mammary gland may comprise the progeny from a single cell.. Development.

[14] Li L, Xie T (2005). Stem cell niche: structure and function.. Annu Rev Cell Dev Biol.

[15] Boulanger CA, Mack DL, Booth BW, Smith GH (2007). Interaction with the mammary microenvironment redirects spermatogenic cell fate in vivo.. Proc Natl Acad Sci U S A.

[16] Booth BW, Mack DL, Androutsellis-Theotokis A, McKay RD, Boulanger CA (2008). The mammary microenvironment alters the differentiation repertoire of neural stem cells.. Proc Natl Acad Sci U S A.

[17] Boulanger CA, Smith GH (2009). Reprogramming cell fates in the mammary microenvironment.. Cell Cycle.

[18] Pouton CW, Haynes JM (2007). Embryonic stem cells as a source of models for drug discovery.. Nat Rev Drug Discov.

[19] Lensch MW, Daheron L, Schlaeger TM (2006). Pluripotent stem cells and their niches.. Stem Cell Rev.

[20] Welm BE, Tepera SB, Venezia T, Graubert TA, Rosen JM (2002). Sca-1(pos) cells in the mouse mammary gland represent an enriched progenitor cell population.. Dev Biol.

[21] Jiang S, Fu Y, Williams J, Wood J, Pandarinathan L (2007). Expression and function of cannabinoid receptors CB1 and CB2 and their cognate cannabinoid ligands in murine embryonic stem cells.. PLoS ONE.

